# Resurrection of *Anolis ustus* Cope, 1864 from synonymy with *Anolis sericeus* Hallowell, 1856 (Squamata, Dactyloidae)

**DOI:** 10.3897/zookeys.619.9650

**Published:** 2016-09-27

**Authors:** José Daniel Lara-Tufiño, Adrián Nieto-Montes de Oca, Aurelio Ramírez-Bautista, Levi N. Gray

**Affiliations:** 1 Laboratorio de Ecología de Poblaciones, Centro de Investigaciones Biológicas, Universidad Autónoma del Estado de Hidalgo. Apartado postal 1-69, Plaza Juárez, 42001 Pachuca, Hidalgo, México; 2 Laboratorio de Herpetología, Departamento de Biología Evolutiva, Facultad de Ciencias, Universidad Nacional Autónoma de México. Circuito exterior s/n, Ciudad Universitaria, 04510 México, Ciudad de México, México; 3 Department of Biology and Museum of Southwestern Biology, University of New Mexico, Albuquerque, New Mexico 87131, United States

**Keywords:** Anolis, Atlantic versant, dewlap, hemipenes, Yucatan Peninsula

## Abstract

In this study, based on a morphological analysis, the resurrection of the name *Anolis ustus* Cope 1864, is proposed for populations from the Yucatán Peninsula (Campeche, Yucatán, and Quintana Roo, Mexico, and Belize), formerly referred as *Anolis sericeus* Hallowell, 1856. *Anolis ustus* differs from *Anolis sericeus* by its mean snout-vent length and number of gorgetal scales in males, in tibia length and head width in females, and dorsal and ventral scales for both sexes. In addition, *Anolis ustus* has a small dewlap of similar size between males and females, whereas in *Anolis sericeus* males have a dewlap much larger than that of the females. These characteristics allow *Anolis ustus* to be identified within the *Anolis sericeus* complex. In this study, a description of the characteristics of the hemipenis is also provided, and its importance in the taxonomy of *Anolis* is discussed.

## Introduction

The name *Anolis sericeus* has a relatively old and complex taxonomic history. *Anolis sericeus* was described by Hallowell (1856) on the basis of a specimen from “El Euceros, Jalapa, Veracruz” (because there is no known location by this name currently, we believe the actual location may be El Lencero, Xalapa, Veracruz). Unfortunately, the type specimen is now also lost (Barbour 1934; Stuart 1963). During the rest of the 19^th^ and the first half of the 20^th^ centuries, several other nominal species of *Anolis* were described from Mexico and Central America (*Anolis sallaei* Günther 1859, *Anolis longicauda* Hallowell 1861, *Anolis cumingii* Peters 1863, *Anolis ustus* Cope 1864, *Anolis heliactin* Cope 1864, *Anolis jacobi* Bocourt 1873, *Anolis kidderi* Ruthven 1933, and *Anolis ustus wellbornae* Ahl 1940), but all of these taxa were eventually placed in the synonymy of *Anolis sericeus* by Boulenger (1885), Barbour (1934), Smith and Taylor (1950), Stuart (1955), Duellman (1961), and Lee (1980). Thus, *Anolis sericeus* has been regarded until recently as a single species with a wide distribution extending from Tamaulipas and Oaxaca on the Atlantic and Pacific versants of Mexico, respectively, to the east (including the Isthmus of Tehuantepec and the Yucatan Peninsula) and south through Central America to Costa Rica (Lee 1980).

Köhler and Vesely (2010) recently proposed that *Anolis sericeus* is actually composed of three species: *Anolis sericeus*, distributed on the Atlantic versant from Tamaulipas, San Luis Potosí, and Hidalgo in Mexico south and east through the Yucatan Peninsula to Belize and Guatemala; *Anolis wellbornae*, from the Pacific versant of Nuclear Central America (Pacific versant of Guatemala to approximately Mazatenango, El Salvador, extreme southern Honduras, and northwestern Nicaragua); and *Anolis unilobatus*, which ranges on the Pacific versant from Oaxaca, Mexico south and east through Guatemala, Honduras, and Nicaragua to Costa Rica. Köhler and Vesely (2010) diagnosed *Anolis sericeus* by the presence of large bilobate hemipenes and dewlaps of similar size (≤ 50 mm^2^) in males and females.

Nonetheless, preliminary observations of substantial geographic variation in several morphological characters (e.g., dewlap size, hemipenial morphology, and numbers of dorsal and ventral scales) among populations of *Anolis sericeus* *sensu* Köhler and Vesely (2010) suggested that this taxon may be composed of more than one species. Herein, we performed a morphological analysis of 140 specimens from throughout the geographic distribution of *Anolis sericeus* to assess the existence of multiple species within this taxon.

## Materials and methods

**Figure 1.**
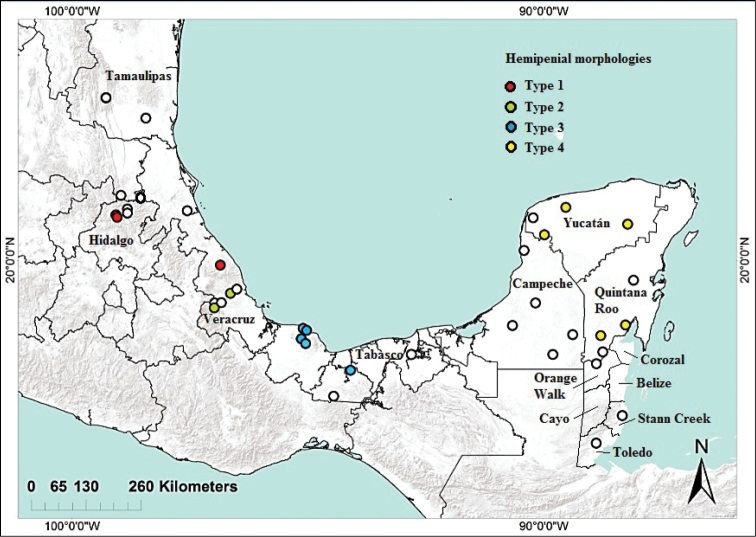
Localities of specimens examined in this study. Colored circles represent specimens with everted hemipenis (see text for details). Areas above 500 m shaded gray.

We examined specimens from most of the geographic distribution of *Anolis sericeus* *sensu* Köhler and Vesely (2010), including specimens from Tamaulipas, Hidalgo, Veracruz, Tabasco, Campeche, Yucatán, and Quintana Roo, Mexico, as well as the two syntypes of *Anolis ustus* in the Natural History Museum of London (NHM 1946.8.5.60-61) and other specimens from Belize (Fig. 1). We performed fieldwork in the states of Hidalgo, Veracruz (north, center and south), and Quintana Roo, Mexico from April to November 2015. Only adult specimens (≥ 38 mm snout-vent lengths [SVL]) were collected. Specimens of *Anolis sericeus* were not found at the type-locality of El Lencero, Xalapa, Veracruz; however, we collected specimens of *Anolis sericeus* at Xotla, Veracruz, only 26 km from the type-locality. Collected specimens were fixed with 10% buffered formalin and preserved in 70% ethanol. Hemipenis were everted and hardened by immersion in formalin for 40 seconds.

The remaining examined specimens were borrowed from the following collections: **ECOSUR**, Chetumal, Quintana Roo (**ECOCHH**); Centro de Investigaciones Biológicas, Universidad Autónoma del Estado de Hidalgo
(CIB); Museo de Zoología Alfonso L. Herrera, Facultad de Ciencias, Universidad Nacional Autónoma de México
(MZFC); Instituto de Investigaciones Biológicas, Universidad Veracruzana
(IIB-UV); Instituto Tecnológico de Ciudad Victoria, Tamaulipas (ITCV); Estación de Biología Tropical de Los Tuxtlas, Veracruz (CAR-EBTT); University of Kansas (KU); Carnegie Museum of Natural History
(CMNH); and the The Natural History Museum, London
(NHM).

Measurements were taken under a microscope trademark Leica (model MS-512X) with a digital caliper trademark Mitutoyo (model CD-8”CX) and recorded to the nearest 0.1 mm. Nomenclature of measured characters follows Köhler (2014). A total of 14 morphometric and 14 meristic characters was recorded in both males and females:

SVL
 snout-vent lenght 


TL
 tail length 


HL
 head length 


HW
 head width 


SL
 snout length 


LDIS
 longitudinal diameter of the interparietal scale 


TDIS
 transverse diameter of the interparietal scale 


NRL
 naris-rostrum length 


IL
 internarial length 


DA
 dewlap area 


AGL
 axilla-groin length 


FL
 forearm length 


ShL
 shank length 


LFT
 length of the fourth toe 


DLW
 dilated lamellae width 


NPS
 number of postrostral scales 


NIS
 number of internasal scales 


NSSSS
 number of scales separating supraorbital semicircles 


NLS
 number of loreal scales 


NSuperciliary
 number of superciliary scales 


NSupraoculars
 number of supraocular scales 


NSupralabials
 number of supralabial scales 


NInfralabials
 number of infralabial scales 


NPS
 number of postmental scales 


NLFT
 number of subdigital lamellae of fourth toe (from the lamella situated at the level of the joint of phalanges III and IV to the beginning of the terminal lamellae 


NTL
 number of terminal lamellae 


NDS
 number of dorsal scales 


NVS
 number of ventral scales 


In addition, the number of gorgetal scales (NGS) in males was recorded. In this review variation in color pattern was not considered because of changes in pattern with preservation. Dewlap area was measured on field specimens only. The area was measured by tracing the outline of the extended dewlap onto a sheet of grid paper with 1 mm^2^ squares, and counted the number of squares that were completely within the outline. Nomenclature for hemipenial structures follows Myers (1971) and Myers et al. (1993). In this study we adopted the unified concept of species proposed by de Queiroz (2007), which states that a species is a metapopulation lineage evolving independently of other lineages.

### Analysis of data

Each examined character was tested for normality with a Komolgorov-Smirnov test using the statistics program STATISTICA v. 7 (StatSoft 2004). To assess whether populations of *Anolis sericeus* comprise multiple morphologically distinct groups we performed a Principal Component Analysis (PCA) of all of the measured characters for each sex; this analysis also was performed to identify characters highly correlated (< -0.50 or > 0.50) with the three main principal components. A Generalized Discriminant Analysis (GDA) was then used with the latter characters, also for each sex, using STATISTICA v. 7. This analysis evaluated whether groups observed in the PCA for both sexes are significantly different using the squared Mahalanobis distances, with an F test (Johnson 2000; Montanero-Fernandez 2015). Wilks’ Lambda test was also used to identify the characteristics that allow discrimination between groups. In addition, when two or more discriminant functions were obtained, an ordination scatter plot was made. If only one function was obtained, a box plot was generated. These graphs were made with the program PAST v. 3.08 (Hammer 2015).

## Results

**Figure 2.**
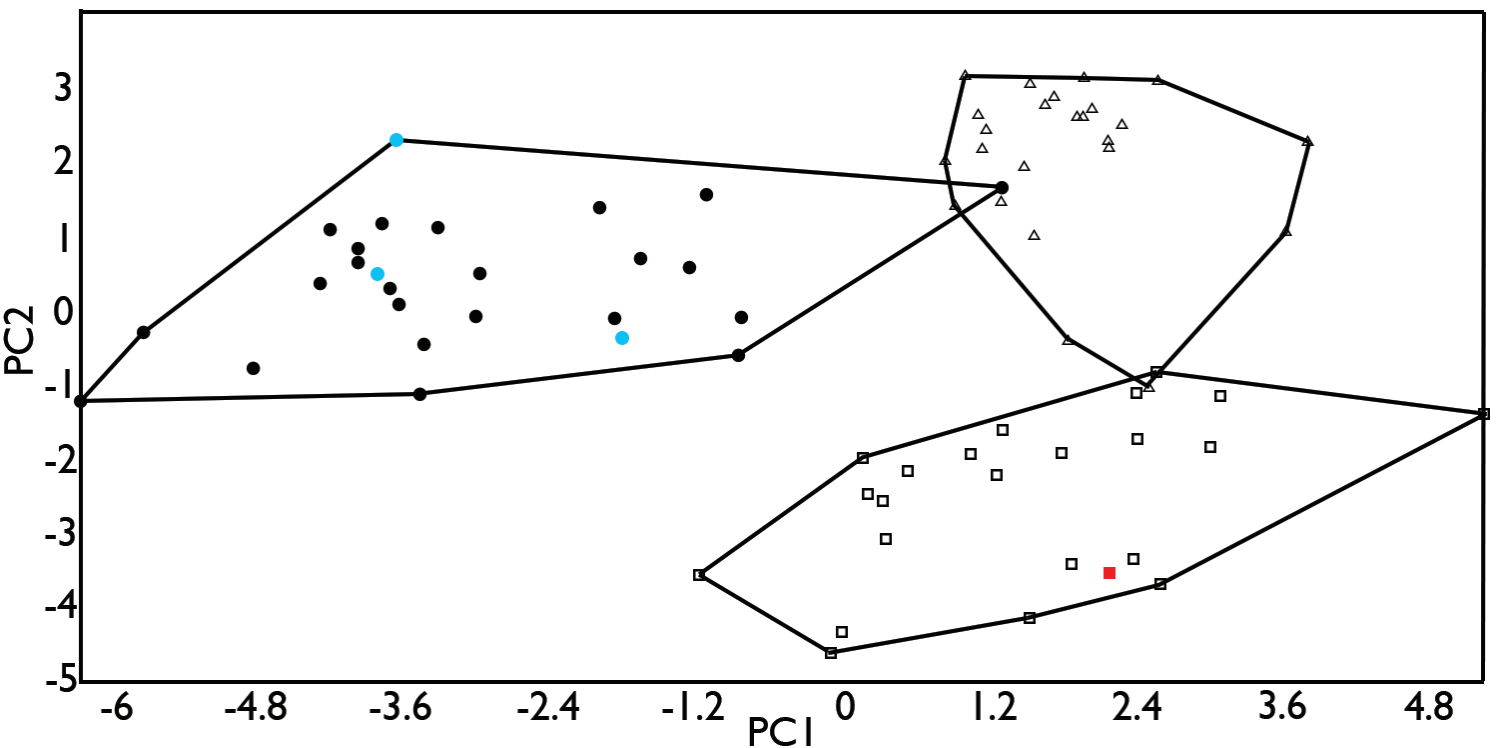
Ordination diagram of principal components 1 and 2 from the PCA of morphological data for males. Circles represent specimens from Tamaulipas, Hidalgo, and northern and central Veracruz; triangles represent specimens from southern Veracruz and western Tabasco; squares represent specimens from the Yucatan Peninsula. Blue circles represent specimens from near the type locality of *Anolis sericeus*. The red square indicates the syntype specimen of *Anolis ustus*.

**Figure 3.**
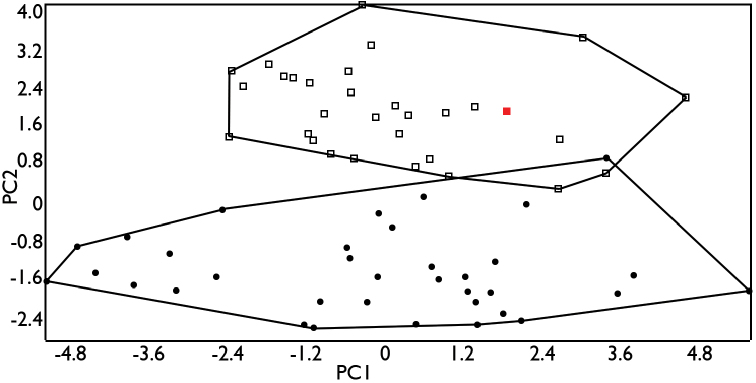
Ordination diagram of principal components 1 and 2 from the PCA of morphological data for females. Circles represent specimens from the Atlantic versant; squares represent specimens from the Yucatan Peninsula. The red square indicates the syntype of *Anolis ustus*.

In the PCA for males, the first three principal components explained 47.11% of the variation in the analyzed characters, of which 18 were highly correlated with these components (Table 1). The PC1 vs PC2 ordination plot (Fig. 2) identified three groups, composed of the specimens from: (*i*) Tamaulipas, Hidalgo, and northern and central Veracruz (i.e., the northern and central portions of the Atlantic versant, or AV1), (*ii*) southern Veracruz and western Tabasco (i.e., the southern portion of the Atlantic versant, or AV2), and (*iii*) Campeche, Yucatán, Quintana Roo, and Belize (i.e., the Yucatan Peninsula, or YP). In the PCA for females, the first three principal components explained 39.58% of the variation in the analyzed characters, of which 13 were highly correlated with these components (Table 1). The PC1 vs PC2 ordination plot identified two morphologically distinct groups, composed of the specimens from: (*i*) Tamaulipas, Hidalgo, Veracruz and western Tabasco (i.e., the Atlantic versant, or AV), and (*ii*) Campeche, Yucatán, Quintana Roo, and Belize (i.e., the Yucatan Peninsula, or YP), respectively (Fig. 3).

**Table 1.** Statistics from the PCA for males and females. Correlation coefficients among characters for the first three principal components. * Characters that explained the highest percentage of variation for each component. Also shown here are the eigenvalue, explained variance, and accumulated explained variance for the first three components.

Characters	Males			Females		
	PC1	PC2	PC3	PC1	PC2	PC3
SVL	-0.85173*	-0.20534	0.07991	-0.85924*	-0.11391	0.06696
TL	-0.63747*	0.42139	0.14205	-0.08738	0.10816	-0.15747
HL	-0.75132*	-0.21300	0.02761	-0.51013*	-0.11974	-0.10871
HW	-0.89301*	-0.01137	0.08725	-0.85500*	0.11310	-0.03773
SL	-0.16205	-0.76203*	0.06875	-0.62754*	-0.47109	-0.06681
LDIS	0.02718	-0.38966	-0.41556	-0.18201	-0.09325	-0.00660
TDIS	-0.26775	-0.34381	-0.40685	0.03118	-0.37480	0.07133
NRL	-0.59741*	-0.35489	-0.15510	-0.37018	0.16997	-0.64634*
IL	-0.53550*	-0.21165	-0.22670	-0.17989	0.16189	-0.63214*
AGL	-0.61223*	-0.32925	0.15488	-0.79493*	-0.22509	0.24664
FL	-0.67579*	0.15599	-0.10419	-0.73863*	0.25637	0.21445
ShL	-0.79747*	0.35656	-0.10989	-0.61786*	0.51700	0.30123
NLFT	-0.40566	-0.09677	-0.16376	-0.39755	0.25495	0.36898
DLW	-0.49120	-0.06745	0.20022	-0.35303	0.03498	-0.11668
NPS	-0.55101*	0.07934	0.26717	-0.19525	0.52569*	-0.29213
NIS	-0.62932*	-0.00681	0.34277	-0.48324	0.12972	-0.56355
NSSSS	0.21946	0.18801	0.61755*	0.10245	0.22969	-0.07205
NLS	-0.24362	0.11200	0.66801*	-0.23420	0.34496	0.35073
NESupraoculars	-0.25903	0.03750	0.14125	-0.42631	0.27921	-0.05066
NSuperciliary	0.24871	-0.46196	0.08580	0.19944	-0.39344	-0.41732
NSupralabials	-0.3210	-0.44249	0.04213	-0.18859	-0.37790	0.42922
NInfralabials	-0.29243	-0.47001	-0.05932	-0.34863	-0.26685	0.06765
NPS	0.07640	0.10760	-0.04845	-0.09248	0.30053	-0.38155
NLFT	0.38912	0.35547	0.46492	0.20960	0.44236	0.20140
NTL	-0.26142	-0.22149	-0.09782	-0.05959	-0.07437	-0.47491
NDS	0.08872	-0.70684*	0.25048	-0.31800	-0.61508*	0.00925
NVS	-0.02688	-0.66557*	0.44827	-0.15787	-0.69712*	-0.15984
DA	-0.52824	0.76774*	-0.11198	-0.09344	-0.67078*	-0.02167
NGS	-0.47798	0.78351*	-0.11481	-	-	-
**Eigenvalue**	7.00	4.58	2.13	5.08	3.45	2.55
**Explained variance (%)**	24.05	15.80	7.35	18.15	12.30	9.11
**Accumulated variance (%)**	24.05	39.80	47.20	18.15	30.45	39.58

**Figure 4.**
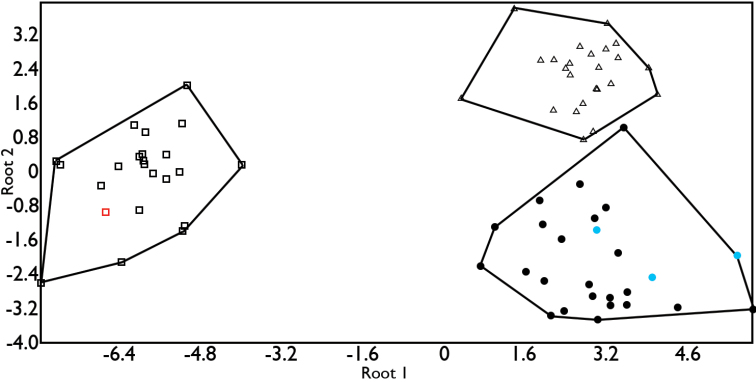
Ordination diagram from the GDA analysis of the morphological data for males. Circles represent specimens from Tamaulipas, Hidalgo, and northern and central Veracruz; triangles represent individuals from southern Veracruz and western Tabasco; squares represent individuals from the YP. Blue circles indicate specimens collected near the type locality of *Anolis sericeus*. The red square indicates the syntype of *Anolis ustus*.

**Figure 5.**
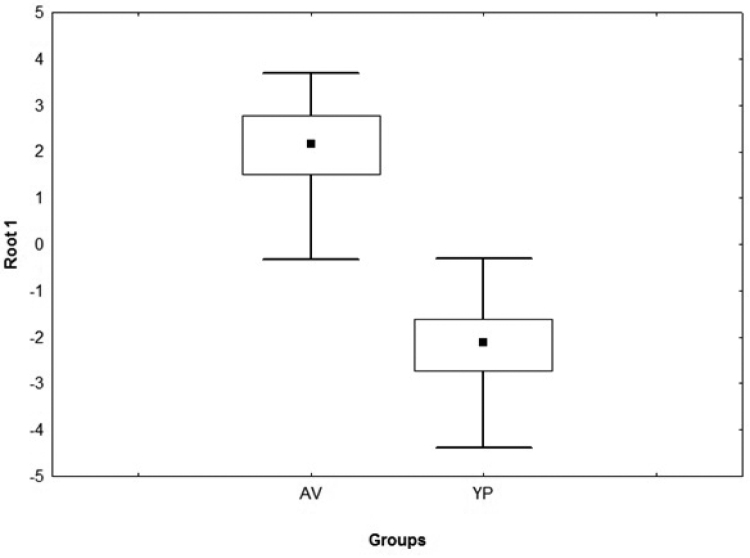
Box plot generated with the root 1 of GDA of morphological data for females. Rectangles: 25–75% from the data; squares: means. Boxplot stems represented 95% confidence interval.

GDA for males showed that the first two functions explained 100% of the total variance; Wilks’s lambda test indicated that SVL, SL, NGS, and DA are the characters that allow discrimination among groups (Table 2). In the ordination scatter plot from this analysis (Fig. 4), individuals of the AV1 and AV2 groups (see above) formed distinct groups that were close to each other, whereas individuals of the YP group formed a third group clearly separated from the other two. The three groups were significantly different from each other (AV1 vs AV2: F = 9.99, p = < 0.0001; AV1 vs. PY: F = 24.79, p = < 0.0001; AV2 vs. YP: F = 17.86, p = < 0.0001). The three specimens from near the type-locality of *Anolis sericeus* belonged to the AV1 group, whereas the male syntype of *Anolis ustus* belonged to the YP group. In the GDA for females, the first function with its own root of 5.26 explained 100% of the total variance, and according to Wilks’s lambda test, ShL, NDS, NVS, and DA are the characters that allow discrimination between groups (Table 3). The AV and YP groups were significantly different (F = 19.37, p < 0.0001). In the box plot the two groups were nearly completely separated (Fig. 5). Descriptive statistics of the characters most important according to the Wilks´s Lambda test for males and females are given in Table 4.

**Table 2.** The Own Root and Explained Variance of canonical functions 1 and 2, and results from Wilks’ Lambda tests from GDA of morphological data for males. Only statistically significant morphological variables according to Wilks’s Lambda test are presented. * *p* < 0.05.

Character	Canonical function 1	Canonical function 2	Wilk’s Lambda	F	p
SVL	-0.0656	-0.2333	0.8896	3.2877	0.0450*
TL	0.0314	-0.0151	0.9573	1.1809	0.3149
HL	0.0138	-0.4059	0.9462	1.5055	0.2312
HW	-0.0147	0.4540	0.9895	0.2802	0.7567
SL	-0.3391	-1.6851	0.7992	6.6572	0.0026*
NRL	-0.7577	0.6368	0.9740	0.7063	0.4980
IL	-0.3024	-1.7315	0.9075	2.6980	0.0765
AGL	-0.0966	0.0590	0.9862	0.3685	0.6934
FL	0.2231	-0.0495	0.9705	0.8054	0.4522
ShL	-0.1509	-0.2002	0.9862	0.3694	0.6928
NPS	-0.0724	-0.4489	0.9440	1.5692	0.2177
NIS	0.4141	-0.0372	0.9674	0.8914	0.4161
NSSSS	-0.1466	0.3262	0.9809	0.5159	0.5998
NLS	-0.1596	-0.3107	0.9710	0.7903	0.4589
NDS	-0.0333	-0.0710	0.9014	2.8966	0.0639
NVS	0.0386	0.0568	0.9606	1.0861	0.3449
NGS	-0.1724	-0.7870	0.8599	4.3144	0.0183*
DA	0.1726	0.0445	0.3439	50.5422	0.0000*
**Own Root**	17.0669	3.4759	–	–	–
**Explained Variance (%)**	0.8308	1.0000	–	–	–

**Table 3.** The Own Root and Explained Variance of canonical function 1 for female morphological data obtained with GDA. Only statistically significant morphological variables according to Wilks’s Lambda test are presented. * *p* < 0.05.

Character	Canonical Function 1	Wilk’s Lambda	F	p
SVL	-0.12862	0.959861	2.21631	0.142485
LCA	-0.07654	0.992848	0.38178	0.539296
WH	1.91975	0.818575	11.74666	0.001186*
LH	-0.74607	0.941677	3.28255	0.075687
LNR	-1.27415	0.972540	1.49646	0.226628
LEN	0.76652	0.970452	1.61375	0.209514
AGL	-0.00895	0.999859	0.00747	0.931467
FL	-0.01099	0.999978	0.00118	0.972746
ShL	0.84336	0.839738	10.11490	0.002458*
NPS	0.14725	0.985193	0.79654	0.376163
NDS	-0.06274	0.926137	4.22696	0.044726*
NVS	-0.09454	0.879576	7.25631	0.009440*
DA	-0.09182	0.737254	18.88838	0.000063*
**Own Root**	4.75	_	_	_
**Accumulated Variance (%)**	100	_	_	_

**Table 4.** Mean ± standard deviation and range for the most important morphological characters according to the GDA.

Character	Males			Females	
	AV1 (*n* = 26)	AV2 (*n* = 24)	YP (*n* = 23)	AV (*n* = 35)	YP (*n* = 32)
**SVL**	45.72 ± 2.71 (38.52–49.94)	39.49 ± 1.32 (36.5–42.38)	41.50 ± 2.20 (37.36–44.59)	41.33 ± 2.48 (38.21–53.01)	44.28 ± 3.8 (35.07–48.93)
**SL**	5.3 ± 0.39 (4.47–6.02)	4.56 ± 0.29 (3.99–5.85)	5.7 ± 0.44 (5.0–6.93)	5.14 ± 0.46 (4.16–6.08)	5.56 ± 0.50 (4.83.6.56)
**ShL**	10.79 ± 0.95 (9.9–13.38)	9.52 ± 0.57 (8.52–10.78)	8.47 ± 0.49 (7.54–9.78)	10.0 ± 1.0 (8.32–11.42)	8.7 ± 0.6 (7.22–9.91)
**NDS**	48.92 ± 6.55 (36–62)	43.59 ± 6.09 (37–60)	57.5 ± 7.60 (44–78)	47.5 ± 7.62 (37–60)	56.7 ± 5.66 (45–71)
**NVS**	42.15 ± 6.14 (32–54)	38.59 ± 5.24 (31–51)	47.7 ± 4.55 (39–59)	39.4 ± 6.11 (28–47)	48.3 ± 6.72 (37–66)
**DA**	97.3 ± 8.12 (85–120)	92.13 ± 5.36 (83–110)	45 ± 5.9 (31–58)	30.14 ± 4.8 (31–40)	41.1 ± 5.1 (32–50)
**NGS**	8.53 ± 0.58 (8–9)	8.31 ± 0.47 (8–9)	5.4 ± 0.61 (4–6)	-	-

### Hemipenial morphology

**Figure 6.**
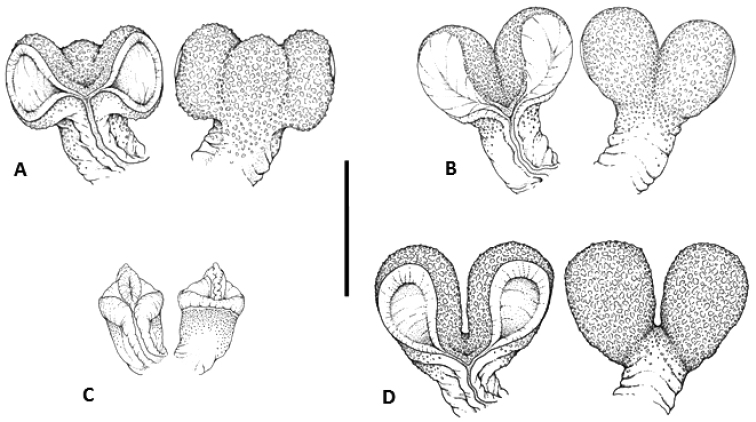
Hemipenial morphologies in sulcate and asulcate views. **A** North of Sierra de Chiconquiaco, Veracruz (AV; CIB 4945) **B** South of Sierra de Chiconquiaco, Veracruz (AV;MZFC 227) **C** Southern Veracruz (AV;CIB 4957) **D** Yucatan Peninsula (CIB 4982). Scale bar: 0.5 mm.

Forty out of 78 examined males had everted hemipenes (29 from the AV and 11 from the YP). Four different hemipenial morphologies were found. The geographic distribution of these morphologies is shown in Fig. 1. The first morphology was found in males from north of the Sierra de Chiconquiaco, Veracruz (n = 9). The hemipenes are large, slightly bilobate with a small protuberance between the lobes; the surface is calyculate, especially in asulcate view. The lobes are well developed, about as wide as the trunk. The borders of the sulcus spermaticus are well developed (Fig. 6A). The second morphology was exhibited by males from south of the Sierra de Chiconquiaco, Veracruz (n = 5). The hemipenes are large, strongly bilobate with no protuberance between the lobes; the surface is conspicuously calyculate in asulcate view. The lobes are well developed, as wide as the trunk, which is relatively long. The borders of the sulcus spermaticus are barely engrossed (Fig. 6B). The third morphological class was exhibited by males from southeast Veracruz (n = 15). The hemipenes are small and unilobate, with a widened crest at the apex. The surface is calyculate, especially near the trunk in asulcate view. The borders of the sulcus spermaticus are well developed (Fig. 6C). The fourth morphology was found in males of the YP group (n = 11). The hemipenes are large, with well-developed lobes larger than the trunk; the surface is strongly calyculate in asulcate view. The borders of the sulcus spermaticus borders are conspicuously developed (Fig. 6D).

## Discussion

Statistical analyses showed three morphologically different groups of males (AV1, AV2, and YP), although only two of females (AV and YP). However, we consider that the morphological evidence that separates the two groups of males from the AV is not enough to question their conspecificity, because they only differ in average SVL and SL from those of the group AV1 (Table 4). At this point, additional evidence supporting their status as distinct species (e.g., molecular data, ecology) is lacking.

**Figure 7.**
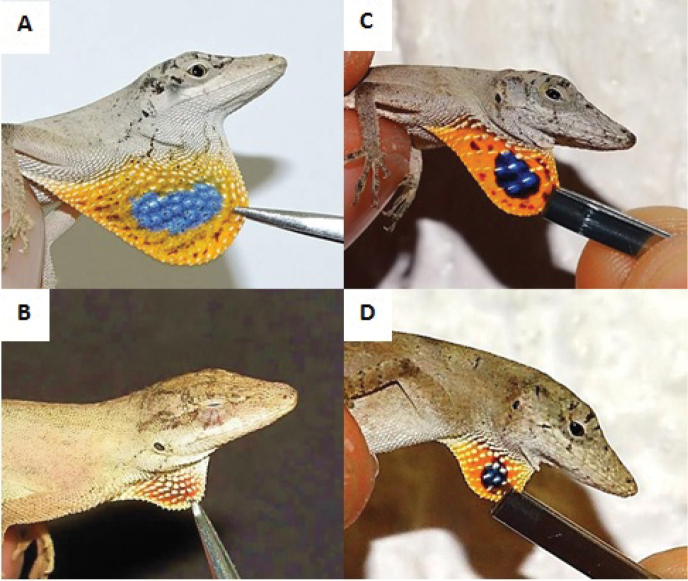
Dewlaps. **A** male from AV, photo by Ismael Reaño Hernández **B** female from AV, photo by Luis M. Badillo Saldaña **C** male from YP **D** female from YP.

The YP group was distinguishable from the AV groups in both males and females. The characters with the largest contributions to the separation of the AV and YP groups included SVL, SL, and NGE in males; HW and ShL in females, and DA, NDS, and NVS in both sexes. Of these characters, the one with the largest contribution to the separation of the AV and YP groups was dewlap size (DA). The differences in dewlap size between males and females of both groups were obvious (Fig. 7). In the AV populations, the dewlap is large in males (> 85 mm^2^, Fig. 7A) and small in females (< 55 mm^2^, Fig. 7B), whereas in the YP populations the males have a much smaller dewlap, only slightly larger than that of females (< 55 mm^2^ and < 50 mm^2^, respectively; Fig. 7C, D). The small size of the dewlap in YP males (and the lack of strong sexual dimorphism in this character) also distinguishes the YP populations from those of *Anolis wellbornae* and *Anolis unilobatus* (Köhler and Vesely 2010). The dewlap does not seem to differ in color between the AV and YP populations; however, the central blue spot is conspicuous in females from the YP (Fig. 7D) and diffuse or absent in females from the AV (Fig. 7B). Two other characters that had an important contribution to the separation of the AV and YP groups were NDS and NVS. In general, males and females of the YP group had more dorsal and ventral scales than those of the AV group (Table 4).

The dewlap has been regarded as a taxonomically important character in *Anolis* by many authors (summarized in Losos 2009). Variation in dewlap size, shape, and color determines many intra- and interspecific interactions in *Anolis*. Thus, the dewlap is an important attribute in the defense of territory and sexual displays. Moreover, it is a primary source of recognition among species (Jenssen 1977; Fitch and Hillis 1984; Losos 2009). It has also been suggested that evolution of the dewlap has been driven by sexual selection (Fitch and Hillis 1984). Therefore, variation in dewlap size and color is likely important for the development of reproductive isolation barriers between populations of the AV and YP groups of *Anolis sericeus*. Lee (1980) compared the dewlap of 25 males and 25 females of *Anolis sericeus* from the YP with that of the male holotype of *Anolis kidderi* Ruthven 1933 and one female referred by Smith (1938) to this species (failing to find significant differences), but did not compare males or females of the AV and YP. Also, Köhler and Vesely (2010) stated that the dewlap of male *Anolis sericeus* is small (< 50 mm^2^) and similar in size to that of females in populations of both the AV and YP. However, we found this statement to be true only for the populations of the YP group.

Male *Anolis sericeus* from the AV and YP groups also differed in hemipenial morphology (Fig. 6). This finding is unsurprising, as it has been noted that hemipenes evolve much faster than other morphological characters in *Anolis* (Klaczko et al. 2015). Given these recent findings, differentiation in hemipenial traits is likely to occur prior to speciation. We suspect many species will be polymorphic for these characters, and using them to delimit species should be done with care. Our own work on this species complex supports this suggestion, as we found at least two different hemipenial morphologies (unilobate and bilobate) within *Anolis sericeus*. Evidence for reproductive isolation between closely related populations with differentiated hemipenes in *Anolis* is currently lacking. For instance, Köhler et al. (2012) found that individuals of *Anolis polylepis*, *Anolis osa*, and putative hybrids between these two taxa were able to copulate and produce offspring even though they possessed differentiated hemipenes. Thus, hemipenes should be treated like any other potentially informative trait that is expected to be varying within species. In addition, recent molecular studies have shown that evolutionary lineages may not be at all concordant with the distributions of forms associated with hemipenes (e.g., Phillips et al. 2015; Gray unpublished).

Although differences in hemipenial morphology between populations may not warrant recognition of the differentiated populations as distinct species (see above), we argue that the other consistent morphological differences between the AV and YP groups of *Anolis sericeus* do warrant their recognition as distinct evolutionary lineages. Because the type locality of *Anolis sericeus* is in Veracruz (El Lencero, Xalapa), the lineage in Tamaulipas, Hidalgo, Querétaro, Veracruz, and Tabasco should retain this name, whereas the oldest available name for the lineage in Campeche, Yucatán, Quintana Roo, and Belize is *Anolis ustus* Cope 1856 (type locality = “Belize”). *Anolis kidderi* Ruthven 1933, described from “Quinta, Mérida, Yucatán” becomes a junior synonym of *Anolis ustus*.

The specimens from Tabasco (two males and two females) were placed within the AV group with the specimens from Veracruz, Hidalgo, and Tamaulipas in our analyses, and possessed the diagnostic characters of this group: the dewlap is large in males and small in females, and both males and females have low counts of NDS and NVS. In contrast, and despite the geographic proximity between their localities and those of the specimens from Tabasco, the specimens from Campeche (seven males and nine females) belonged into the YP group and possessed the diagnostic characters of this group: the dewlap is small in both males and females and the NDS and NVS counts are high. Recent field work in southern Campeche, between the coordinates 18°07'38.67"N, 91°36'43.91"W and 18°22'48.60"N, 91°11'54.32"W (WGS84) revealed an abrupt transition in form (LNG, unpublished). While every non-Yucatan population within the *Anolis sericeus* group exhibits strong sexual dimorphism in dewlap size between males and females, *Anolis ustus* stands out as the one lineage that is easily diagnosable morphologically. Additional studies are needed to determine the existence of a contact zone between the two groups, and the existence and extent of any gene flow between them.

There were no evident differences in behavior or microhabitat between the AV and YP groups of *Anolis sericeus*. Individuals of both groups were observed perching between 30 and 400 cm on grasses, branches, or thin trunks. Also, individuals of both groups were found in open areas bordering dense vegetation, pasture land, and crop fields. In our experience, lizards in the *Anolis sericeus* group tend to be quite variable in morphology, habitat preference, and behavior. Lee (1980) found some evidence of local adaptation within populations and follow-up studies are needed to corroborate his findings. Given the extensive environmental variation found within the geographic range of the *Anolis sericeus* group, convergence in some morphological traits (despite divergent evolutionary history and distant geographic proximity) is likely, making species identification exceptionally difficult in this group. *Anolis ustus*, thanks to a small dewlap size in both sexes, appears to be the only lineage that is easily diagnosable within the complex.

## Appendix 1

**Examined specimens**

***Anolis sericeus*.- MÉXICO**: **Tamaulipas**: Victoria: ITCV 0097; González: ITCV 0174. **Hidalgo**: San Felipe Orizatlán: CIB 2799-2800; Jacala: CIB 4944-4949; Molango: CIB-4950; Tepehuacán de Guerrero: CIB-4951; Pisaflores: CIB-4952. **Veracruz**: Tuxpan: IIB-UV 040-044, 047-048, 052, 055-057, 059; Ixhuatlán del Café: MZFC 227; Misantla: MZFC 124-128, 235; Huatusco: MZFC 29996-29997, 29991, 29983, 29988, 2993; Atoyac: MZFC 29994; Uxpanapa: MZFC 1931-1932, 476, 463; Xotla: CIB-4953-4955; San Andrés Tuxtla: MZFC 01789, 01790, 01797, 01811-01812, CIB- 4956-4979; Acayucan:MZFC 01792; Las Choapas: MZFC 0108, 01809. **Tabasco**: Emiliano Zapata: MZFC 241-244. ***Anolis ustus*.- Campeche**: Escárcega: MZFC 245-249, 251, 254; Calakmul: ECOCHH 628, 698, 1283, 1459, 1518, 1526, 1290; Isla Jaina: MZFC 0283. **Quintana Roo**: Othón P. Blanco:MZFC 8690, 01852; Tres Garantías: ECOCHH 2284; La Pantera: ECOCHH 0912; Chetumal: ECOCHH 1557, CIB-4980-4989; Felipe Carrillo Puerto: ECOCHH 1807, 1880; Ejido Caobas: ECOCHH 2578, 2590; Xpujil: ECOCHH 0976; La Unión: ECOCHH 144. **Yucatán**: Mérida: MZFC 258, 286-287, 293, 300, 305; Valladolid: MZFC 316; Celestún: ECOCHH 1675, 1679, 1725. **BELIZE**: “Belize” NHM-1946.8.5.60-61; Toledo: KU 299709; Stann Creek: KU 157228-157229.
